# High Dependency Unit Admissions among Patients with Lower Extremity Long Bone Fracture Visiting the Department of Orthopaedics in a Tertiary Care Centre

**DOI:** 10.31729/jnma.8430

**Published:** 2024-02-29

**Authors:** Satish Prasad Barnawal, Bibek Banskota, Nitesh Raj Pandey, Saroj Rijal, Tarun Rajbhandari, Subhash Regmi, Ashok Kumar Banskota

**Affiliations:** 1 Department of Orthopedic Surgery, B&B Hospital Pvt. Ltd., Gwarko, Lalitpur, Nepal

**Keywords:** *femoral fractures*, *prevalence*, *tibial fractures*, *traffic accidents*

## Abstract

**Introduction:**

Lower extremity long bone, femoral and tibial shaft, fractures often have associated injuries. Patients with lower extremity long bone fractures in the Department of Orthopaedics can land up in high dependency unit admissions, mostly due to underlying complications. The study aimed to find out the prevalence of high dependency unit admissions among patients with lower extremity long bone fractures visiting the Department of Orthopaedics in a tertiary care centre.

**Methods:**

A descriptive cross-sectional study was conducted among patients with lower extremity long bone fractures in a tertiary care centre. The data from 1 March 2017 to 31 January 2020 was collected from the medical records from 1 August 2020 to 30 September 2020. All patients with femoral or tibial shaft fractures in isolation or a part of a multi-system injury were included. Patients with inadequate data were excluded. A convenience sampling method was used. The point estimate was calculated at a 95% Confidence Interval.

**Results:**

Among 507 patients with lower extremity long bone fractures, 137 (27.55%) (23.66-31.44, 95% Confidence Interval) required high dependency unit admission. Among them, 119 (86.86%) were males. A total of 71 (51.82%) cases involved 2-wheelers.

**Conclusions:**

The prevalence of high dependency unit admission among patients with lower extremity long bone fractures was high and majority of them required multidisciplinary approach.

## INTRODUCTION

Road traffic accidents (RTA) are a leading public health problem.^1^ Trauma is a frequent cause of death below forty years of age.^2^ As vehicular traffic increases, the morbidity of severe lower limb injury rises.^3^ This includes fracture of the femur and/or tibia. Lower extremity long bone fractures (LELBF) require a multidisciplinary approach.

Most common fracture sustained is a tibia fracture (26%).^4^ Fat embolism syndrome (FES), acute lower limb ischemia, acute compartment syndrome (ACS), and crush injury are fatal. Head and thoracic injuries are found in 50% and extremity or pelvic fractures in around 30%.^2^ Patients are prone to develop ACS.^5^ Major trauma requires urgent and highly specialised care.^2^

The study aimed to find out the prevalence of high dependency unit admissions among patients with lower extremity long bone fractures visiting the Department of Orthopaedics in a tertiary care centre.

## METHODS

A descriptive cross-sectional study was conducted among patients with lower extremity long bone fractures visiting the Department of Orthopaedics, B&B Hospital Pvt. Ltd., Gwarko, Lalitpur, Nepal. The data from 1 March 2017 to 31 January 2020 was collected from the medical records from 1 August 2020 to 30 September 2020. Ethical approval was obtained from the Institutional Review Committee (Reference number: IRC_2020_07_15-001). All shaft of femur or tibia fractures either in isolation or as a part of a multi-system injury were included in the study. The patients with inadequate data were excluded. A convenience sampling method was used. The sample size was calculated using the following formula:


$$\begin{array}{ll} \text{n} & ={\text{Z}}^{2}\times \frac{\text{p}\times \text{q}}{{\text{e}}^{\text{2}}}\\ & =1.96^{2}\times \frac{0.50\times 0.50}{0.05^{2}}\\ & =385 \end{array}$$

Where,

n = minimum required sample size
Z = 1.96 at 95% Confidence Interval (CI)
p = prevalence taken as 50% for maximum sample size
q = 1-p
e = margin of error, 5%

The calculated sample size was 384. However, we included 516 patients with lower extremity long bone fractures.

The variables used were baseline information, nature of injury and mode of injury along with the occurrence of FES, ACS, acute lower limb ischemia and crush injury. Associated multiple rib fractures, head injuries, spine injuries, pelvis injuries and degloving injuries were also recorded. Diagnosis of tibia, femur and other fractures was done based on standard radiographic evaluation. Assessment of FES, ACS, lower limb ischemia and crush injury was done based on appropriate clinical examination.

The data were entered and analysed using IBM SPSS Statistics version 20.0. The point estimate was calculated at a 95% CI.

## RESULTS

**Figure 1:**
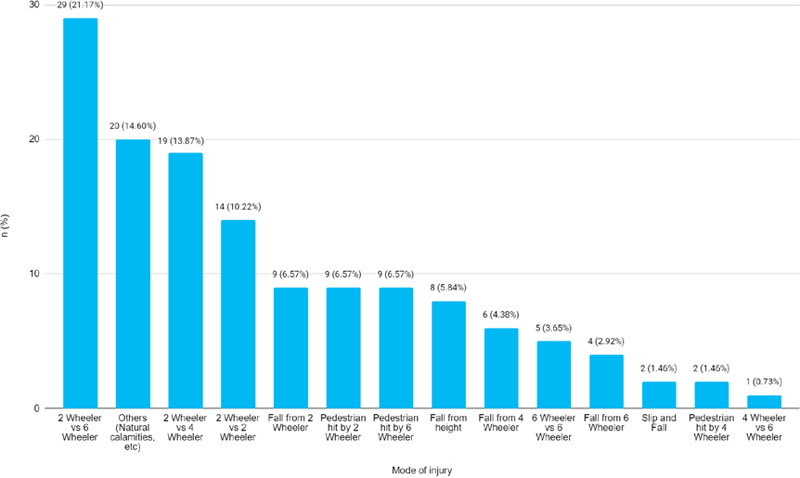
Mode of injury due to which the patients sustained lower extremity long bone fracture (n = 137).

Among 507 patients with lower extremity long bone fractures, 137 (27.55%) (23.66-31.44, 95% CI) required HDU admission. The mean age of patients was 35.23±16.67 years. Among them, 119 (86.86%) were males. A total of 71 (51.82%) cases involved 2-wheelers (Figure 1).

'Others: Unknown, natural calamity, fall of heavy objects over lower extremity, etc.

**Figure 2:**
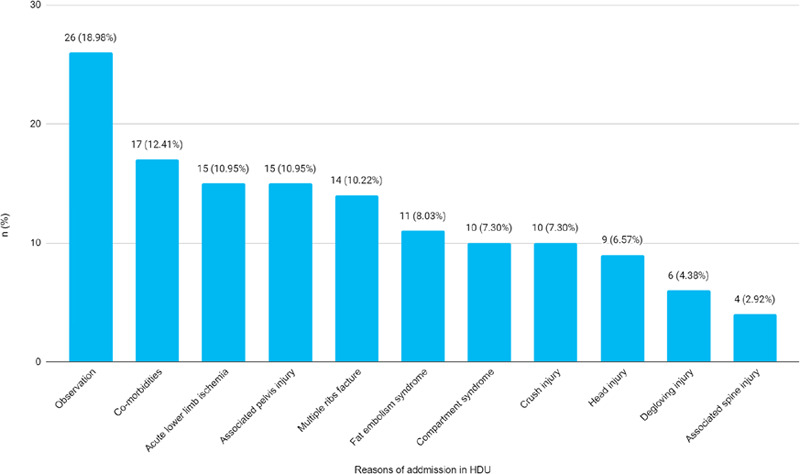
Reasons for admission in HDU among patients sustained lower extremity long bone fracture (n= 137).

A total of 6 (4.38%) patients had expired and 3 (2.19%) patients had left the hospital against medical advice. Among them, 17 (11.64%) patients had comorbidities and 15 (10.95%) had acute lower limb ischemia (Figure 2).

## DISCUSSION

Among 516 patients with lower extremity long bone fractures, 37 (27.55%) required HDU admission with a multidisciplinary approach. This study found that 71 (51.82%) of the RTA involved 2-wheeler vehicles.

Eleven (8.03%) of patients with LELBF in HDU had FES. This finding is consistent with other studies. A study reported the incidence of FES in long bone fractures as 2-5%.^9^ According to a study, 10 cases of fat embolism in Apex Trauma Center ICU in a 6-month period.^6^ Intravasation of fat and medullary contents is found in over 95% of fractures.^10^ But FES occurs in 1-10% of patients in isolated femoral fractures and even more frequently in bilateral fractures.^11^ It can occur within 12-72 hours following traumatic skeletal injuries.^12^ It is described as a triad of pulmonary, central nervous system and skin manifestations.^13^ Respiratory insufficiency is a crucial component of in acute phase.^10^ FES involves multiple organ systems and can cause a devastating clinical deterioration within a few hours.^10^ Early diagnosis and appropriate supportive care reduce complications and mortality.^9^ All such patients require HDU admission.^6^ Mortality is low with modern ICU care.^14^ Mortality has decreased to less than 10%, and in patients who survive most symptoms will resolve.^11^

In this study, 15 (10.95%) of patients with LELBF in HDU had acute lower limb ischemia. It appears higher in comparison to some other reports. A study reported that 7% of patients with upper limb, lower limb and pelvic fractures have vascular damage.^2^ Similarly, peripheral vascular injuries constituted 4-6% of all major traumas in some other reports.^15^ The reason for a higher number of cases of vascular injury in this study could be due to patients, which included the patients with lower limb long bone fractures alone unlike other studies that have reported overall incidence.

Grade IIIC lower limb injuries are at high risk for amputation.^3^ A total of 13.86% of patients with LELBF in HDU underwent amputation. The finding this study is in line with some other studies. Primary amputation was performed in 6 (15%) patients as an initial procedure in a study of 41 cases with grade IIIC lower limb fractures.^3^

Ten (7.30%) of patients with LELBF in HDU had ACS. The finding in this study matches with evidence. A total of 7.73% of all tibial diaphyseal fractures were reported to have ACS in a study.^14^ Similarly, 10.4% of tibial plateau fractures had ACS in a report.^5^ We also had more incidences in similar age groups. Failure to diagnose ACS and treat it in time can lead to catastrophic consequences that are devastating to patients as well as surgeons.^14^ The decision to perform fasciotomy for ACS has to be clinical and liberal use of fasciotomies appears to be associated with lower rates of amputation.^13^

There are a few limitations in this study. It was a retrospective study. The study size was small done in a single centre. There were no definitive criteria for admission to HDU in this resource-limited setup.

## CONCLUSIONS

The prevalence of high dependency unit admission among patients with lower extremity long bone fractures was high and majority of them required multidisciplinary approach. Further research with larger, prospective, multi-center studies is needed to establish definitive admission criteria for high dependency units in resource-limited settings, allowing for more comprehensive insights into the management and outcomes of patients with lower extremity long bone fractures.
